# The effects of so‐tDCS polarity on memory‐related NREM sleep oscillations in healthy older adults

**DOI:** 10.1002/alz70861_108333

**Published:** 2025-12-23

**Authors:** Buse Dikici, Robert Malinowski, Jan‐Bernhard Kordaß, Julia Ladenbauer, Agnes Flöel

**Affiliations:** ^1^ University Medicine Greifswald, Greifswald, Mecklenburg‐Vorpommern Germany

## Abstract

**Background:**

Aging significantly alters sleep architecture, notably reducing cortical slow oscillations (SO; <1Hz) and thalamocortical sleep spindles (8‐15Hz) during non‐rapid‐eye‐movement (NREM) sleep. The application of slow oscillatory direct current stimulation (so‐tDCS) with anodal polarity to increase excitability during SO up‐states has been shown to enhance these NREM sleep oscillations and their coupling, which is relevant for memory consolidation. However, recent findings indicate that global cortical hyperpolarized SO down‐states are essential in triggering thalamic spindles. This suggests that cathodal so‐tDCS, which may facilitate SO down‐states, could prove more effective. As its effects during sleep remain largely unexplored, this study aimed to assess how cathodal so‐tDCS modulates NREM sleep oscillations in healthy older adults.

**Method:**

In a cross‐over design, the effects of anodal and cathodal so‐tDCS on NREM sleep oscillations, including slow oscillations, spindle activity, and their coupling, were compared to sham in 22 healthy elderly participants (55‐79) during a 90‐minute afternoon nap. Additionally, cortical excitation/inhibition (E/I) balance was measured via EEG spectral slope, and chronotype was evaluated as a potential factor influencing inter‐individual variability in stimulation response.

**Result:**

Our results present polarity‐dependent effects, with cathodal so‐tDCS prolonging SO down‐states (F_(2,42)_=6.132, *p* =0.0046; cathodal‐sham: *p* ‐adj=0.015, Cohen’s dz=0.668) and shortening SO up‐states (F_(2,42)_=6.432, *p* =0.004; anodal‐sham *p* ‐adj=0.032, dz=0.56) relative to sham without affecting other SO parameters, spindle activity, or SO‐spindle coupling. In contrast, anodal stimulation improved SO‐spindle synchrony (dz=0.824) and increased spindle power, particularly in individuals with an intermediate‐ to evening‐chronotype compared to sham (r=‐0.59, *p* =0.004; 35.1% variance). Chronotype moderated responsiveness to anodal stimulation. Notably, anodal so‐tDCS increased cortical excitability relative to sham, as indicated by elevated E/I balance (F_(2,42)_=6.121, *p* =0.005; *p* ‐adj=0.004, dz=0.752), while cathodal stimulation did not produce the expected inhibitory shift.

**Conclusion:**

A change in polarity from anodal to cathodal slow oscillatory stimulation does not benefit memory‐related NREM oscillations in healthy older adults. However, our findings suggest that increased cortical excitability may contribute to the oscillatory effects of anodal so‐tDCS. Additionally, chronotype appears to moderate stimulation outcomes, offering insight into personalized interventions.

